# The International Finance Facility for Immunisation: stakeholders’ perspectives

**DOI:** 10.2471/BLT.15.166553

**Published:** 2016-06-28

**Authors:** Tim Crocker-Buque, Sandra Mounier-Jack

**Affiliations:** a London School of Hygiene & Tropical Medicine, 15–17 Tavistock Place, London WC1H 9SH, England.

## Abstract

**Objective:**

To evaluate stakeholders’ understanding and opinions of the International Finance Facility for Immunisation (IFFIm); to identify factors affecting funding levels; and to explore the future use of IFFIm.

**Methods:**

Between July and September 2015, we interviewed 33 individuals from 25 organizations identified as stakeholders in IFFIm. In total 22.5 hours of semi-structured interviews were recorded, transcribed and analysed using a framework method.

**Findings:**

Stakeholders’ understanding of IFFIm’s financing mechanism and its outcomes varied and many stakeholders wanted more information. Participants highlighted that the change in the macro-economic environment following the 2008 financial crisis affected national policy in donor countries and subsequently the number of new commitments IFFIm received. Since Gavi is now seen as a successful and mature organization, participants stated that donors prefer to donate directly to Gavi. The pharmaceutical industry valued IFFIm for providing funding stability and flexibility. Other stakeholders valued IFFIm’s ability to access funds early and enable Gavi to increase vaccine coverage. Overall, stakeholders thought IFFIm was successful, but they had divergent views about IFFIm’s on-going role. Participants listed two issues where bond financing mechanisms may be suitable: emergency preparedness and outcome-based time-limited interventions.

**Conclusion:**

The benefit of pledging funds through IFFIm needs to be re-evaluated. There are potential uses for bond financing to raise funds for other global health issues, but these must be carefully considered against criteria to establish effectiveness, with quantifiable pre-defined outcome indicators to evaluate performance.

## Résumé

**Objectif:**

Évaluer la compréhension et l'opinion qu'ont les parties prenantes sur la Facilité financière internationale pour la vaccination (International Finance Facility for Immunisation / IFFIm); identifier les facteurs qui influent sur les niveaux de financement; et s'interroger sur l'utilisation future de l'IFFIm.

**Méthodes:**

Entre juillet et septembre 2015, nous avons interrogé 33 membres provenant de 25 organisations identifiées comme des parties prenantes de l'IFFIm. Au total, 22,5 heures d'entretiens semi-structurés ont été enregistrées, transcrites et analysées en utilisant une méthode de cadre.

**Résultats:**

La perception par les parties prenantes du mécanisme de financement de l'IFFIm et de ses résultats est variable, et nombre de parties prenantes souhaiteraient avoir plus d'informations. Les participants ont souligné le fait que le changement de l'environnement macroéconomique survenu après la crise financière de 2008 a affecté les politiques nationales dans les pays donateurs et par conséquent le nombre de nouveaux engagements reçus dans le cadre de l'IFFIm. Étant donné que Gavi- L'Alliance du vaccin est aujourd'hui considérée comme une organisation arrivée à maturité et une réussite, les participants ont indiqué que les bailleurs de fonds préfèrent désormais donner directement à Gavi. Pour sa part, l'industrie pharmaceutique apprécie l'IFFIm parce qu’elle permet à la fois une stabilité des financements et de la souplesse. D'autres parties prenantes apprécient le fait que l'IFFIm permette d'accéder rapidement à des fonds et qu'elle permette à Gavi d'améliorer la couverture vaccinale. De manière générale, les parties prenantes estiment que l'IFFIm est un succès, même si leur opinion diverge quant au rôle de l'IFFIm à l’avenir. Les participants ont évoqué deux applications pour lesquelles des mécanismes de financement obligataire pourraient être appropriés: les plans d'intervention d'urgence et des interventions orientées-résultats et limitées dans le temps.

**Conclusion:**

Il est nécessaire de réévaluer les avantages des fonds récoltés par l'IFFIm. Des financements obligataires pourraient être utilisés pour lever des fonds pour d'autres enjeux sanitaires mondiaux. Mais ces utilisations potentielles devront être attentivement considérées au regard de critères permettant de déterminer leur efficacité et en prévoyant des indicateurs de résultats quantifiables pour évaluer leur performance.

## Resumen

**Objetivo:**

Evaluar el conocimiento y las opiniones de los participantes del Fondo Financiero Internacional para la Inmunización (FFII), identificar los factores que afectan los niveles de financiación y explorar el futuro uso del FFII.

**Métodos:**

Entre julio y septiembre de 2015, se entrevistó a 33 individuos de 25 organizaciones identificadas como participantes del FFII. En total, se grabaron, transcribieron y analizaron 22,5 horas de entrevistas semiestructuradas utilizando un método de marco.

**Resultados:**

El conocimiento de los participantes sobre el mecanismo de financiación del FFII y sus resultados varió y muchos de ellos querían más información. Los participantes destacaron que el cambio del entorno macroeconómico tras la crisis financiera de 2008 perjudicó a la política nacional de países donantes y, posteriormente, el número de nuevos compromisos que recibió el FFII. Puesto que ahora la Gavi está considerada como una organización madura y de éxito, los participantes declararon que los donantes prefieren donar directamente a la Gavi. La industria farmacéutica valoró el FFII por ofrecer estabilidad y flexibilidad de financiación. Otros participantes valoraron la capacidad del FFII para tener acceso rápido a los fondos y permitir a la Gavi aumentar la cobertura de la vacunación. En general, los participantes pensaron que el FFII tenía éxito, pero tenían opiniones diferentes sobre el papel actual de dicho Fondo. Enumeraron dos asuntos para los que los mecanismos de financiación de bonos podrían ser adecuados: la preparación de emergencia y las intervenciones en un tiempo limitado basadas en los resultados.

**Conclusión:**

Se deben volver a evaluar los beneficios de otorgar fondos a través del FFII. Existen posibles usos para los que la financiación de bonos aumente los fondos para abordar otros problemas sanitarios globales, pero es necesario analizarlos en profundidad frente a los criterios para establecer la eficacia, con indicadores cuantificables de resultados predefinidos para evaluar el rendimiento.

## ملخص

**الغرض:**

تقييم درجة فهم الجهات المعنية والآراء الخاصة بمرفق التمويل الدولي للتحصين (IFFIm)؛ لتحديد العوامل المؤثرة على مستويات التمويل؛ ولاستكشاف مدى الاستفادة المستقبلية من مرفق التمويل الدولي للتحصين.

**الطريقة:**

قمنا في الفترة ما بين شهري يوليو وسبتمبر من عام 2015 بإجراء مقابلات مع 33 فردًا يمثلون 25 منظمة يتم التعريف عنها باعتبارها جهات معنية في مرفق التمويل الدولي للتحصين. وتم تسجيل ونسخ وتحليل ما مجموعه 22.5 ساعة من المقابلات شبه المقننة باستخدام طريقة العمل النظامي.

**النتائج:**

اختلفت درجة فهم الجهات المعنية لآلية التمويل التابعة لمرفق التمويل الدولي للتحصين والنتائج المترتبة عليها ورغبت العديد من الجهات المعنية في معرفة المزيد من المعلومات. وقام المشاركون بتسليط الضوء على أن التغيير في البيئة الاقتصادية الكلية الذي حدث في أعقاب الأزمة المالية في عام 2008 أثر على السياسة الوطنية في الدول المانحة وبالتالي أثر في عدد الالتزامات الجديدة التي استلمها مرفق التمويل الدولي للتحصين. بما أن منظمة Gavi يتم اعتبارها الآن منظمة ناجحة ومتطورة، فقد أكد المشاركون على أن المتبرعين يفضلون التبرع مباشرة إلى Gavi. قامت صناعة المنتجات الدوائية بتقدير مرفق التمويل الدولي للتحصين لتوفيره عوامل الاستقرار والمرونة في التمويل. كما قامت الجهات المعنية الأخرى بتقدير مرفق التمويل الدولي للتحصين لإمكانية حصوله على الأموال مبكرًا وتمكين منظمة Gavi من زيادة تغطية التلقيح. بشكل عام، اعتقدت الجهات المعنية بأن مرفق التمويل الدولي للتحصين كان ناجحًا، ولكن كانت لديهم آراءً متباينة حول الدور المستمر لمرفق التمويل الدولي للتحصين. قام المشاركون بذكر مشكلتين يمكن أن تعتبر آليات التمويل بموجب السندات مناسبة لحلها وهما: التأهب لحالات الطوارئ والتدخلات المحدودة بالزمن والقائمة على المحصلات.

**الاستنتاج:**

هناك حاجة لإعادة تقييم فائدة التبرع بالأموال من خلال مرفق التمويل الدولي للتحصين. هناك العديد من الاستخدامات المحتملة للتمويل بموجب السندات لجمع الأموال بهدف حل المشكلات الصحية العالمية الأخرى، ولكن ينبغي مراعاة هذه الاستخدامات بعناية مقابل المعايير لتحقيق الفعالية، مع مؤشرات النتائج المحددة مسبقًا والقابلة للقياس لتقييم الأداء.

## 摘要

**目的:**

评价利益相关者对国际免疫融资机制 (IFFIm) 的理解和看法；确定影响资金水平的因素；以及探讨国际免疫融资机制 (IFFIm) 的未来用途。

**方法:**

在 2015 年 7 月至 9 月期间，我们采访了来自 25 个机构的 33 位确认为国际免疫融资机制 (IFFIm) 利益相关者的人员。使用框架法录音、记录和分析了共耗时 22.5 小时的半结构式访谈。

**结果:**

利益相关者对国际免疫融资机制 (IFFIm) 的融资机制和结果的理解各不相同，并且，许多利益相关者希望获得更多信息。参与者强调，2008 年金融危机之后的宏观经济环境变化影响了援助国的国家政策，进而影响了国际免疫融资机制 (IFFIm) 收到的新捐款的数目。由于全球疫苗免疫联盟 (Gavi) 目前被视为一个成功且成熟的机构，参与者表明援助国更愿意直接向全球疫苗免疫联盟 (Gavi) 捐款。制药行业因资金的稳定性和灵活性而非常重视国际免疫融资机制 (IFFIm)。其它利益相关者则重视国际免疫融资机制 (IFFIm) 能够较早获取资金并促使全球疫苗免疫联盟 (Gavi) 增加疫苗覆盖范围的能力。整体来说，利益相关者认为国际免疫融资机制 (IFFIm) 是成功的，但是对其今后的角色，他们意见不一。参与者列出了两处可能需要债券融资机制的问题领域：应急准备和基于结果的限时干预措施。

**结论:**

需要对通过国际免疫融资机制 (IFFIm) 筹集资金的优势进行重新评估。债券融资在其他全球性卫生问题的融资上有潜在用途，但是我们必须根据标准对其进行仔细斟酌以提高效率，并通过可量化的预定义成果指标评估其表现。

## Резюме

**Цель:**

Оценить понимание заинтересованными лицами Международного механизма финансирования иммунизации (ММФИ) и их мнения о нем, определить факторы, влияющие на уровень финансирования, и изучить возможности будущего применения ММФИ.

**Методы:**

В период между июлем и сентябрем 2015 года были проинтервьюированы 33 человека из 25 организаций, определенных в качестве сторон, заинтересованных в ММФИ. В общей сложности было записано, расшифровано и проанализировано с помощью матричного метода 22,5 часа полуструктурированных интервью.

**Результаты:**

Понимание заинтересованными лицами ММФИ и его результатов было неодинаковым, и многим заинтересованным лицам требовалась дополнительная информация. Интервьюируемые отметили, что изменение макроэкономического климата после финансового кризиса 2008 года повлияло на национальную политику в странах-донорах и, как следствие, на количество новых обязательств, полученных ММФИ. Поскольку Gavi в настоящий момент считается успешной и устоявшейся организацией, интервьюируемые заявили, что доноры предпочитают предоставлять средства напрямую этой организации. Представители фармацевтической отрасли отметили полезность ММФИ в плане обеспечения стабильного и гибкого финансирования. Другие заинтересованные лица оценили то, что с помощью ММФИ можно заблаговременно получать доступ к финансам и что благодаря ему Gavi удалось увеличить охват вакцинации. В целом заинтересованные лица признали успешность ММФИ, но расходились во взглядах относительно его текущей роли. Интервьюируемые назвали две сферы, в которых механизмы облигационного финансирования могут быть целесообразны: готовность к чрезвычайным ситуациям и основанные на результатах, ограниченные во времени вмешательства.

**Вывод:**

Необходима повторная оценка преимущества предоставления финансов посредством ММФИ. Облигационное финансирование потенциально может быть использовано для привлечения инвестиций с целью решения других проблем международного здравоохранения, однако для каждого такого случая необходимо рассматривать целесообразность его применения с точки зрения эффективности, рассчитываемой с помощью предварительно устанавливаемых, поддающихся количественному измерению показателей результативности.

## Introduction

Gavi, the Vaccine Alliance, finances vaccine programmes in low-income countries. In 2006, Gavi recognized that to reach high vaccine coverage levels as soon as possible, significantly, more funds were needed than were available. In response, the British Department for International Development, the Gates Foundation, United Nations Children’s Fund and the financial services industry created the independent charity, the International Finance Facility for Immunisation (IFFIm).

**Fig. 1:**
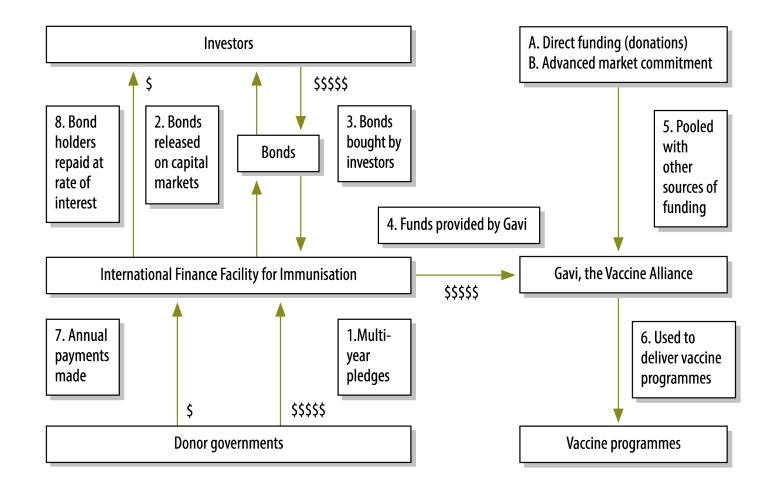
Funding of Gavi, the Vaccine Alliance

Note: The number of dollar signs ($) is an indication of the amount of money that has been transferred.

Between 2000 and 2015 two-thirds of Gavi’s funding – that is, 11.6 billion United States Dollars (US$) – came from donations by governments.^1^ Every five years governments pledge to donate a certain amount and then make regular payments to Gavi. IFFIm enables governments to make a legally binding long-term commitment to IFFIm, for example an annual payment of US$ 20 million for 20 years (Fig. 1), instead of donating directly to Gavi. Next, IFFIm creates bonds – that is, a type of long-term loan – to the value of the total amount committed by governments (in this example US$ 400 million). International investors then buy these bonds, thus immediately providing IFFIm with US$ 400 million. Gavi will have access to these funds by applying to IFFIm. IFFIm pays back bondholders over time with the annual payments from the governments.

The proposed benefit of IFFIm was to make the money from future donations available immediately, so that vaccine programmes could be scaled up to reach the goal of herd immunity earlier. However, there are two costs involved in this financing mechanism. First, the administration costs of IFFIm have been estimated between 4.1% to 4.6% of the pledged amount over the 20-year duration of the current commitments.^2^ Between 2010 and 2014, these costs averaged US$ 115 million per year, with the World Bank acting as treasury manager. The second cost is the payment of interest to bondholders, which is difficult to calculate as it depends on currency and market conditions.

Between 2006 and 2014, IFFIm has received in total US$ 6.5 billion of long-term commitments from 10 donor governments (Table 1) and has raised US$ 5 billion for Gavi through selling bonds (the difference of US$ 1.5 billion is held by IFFIm to reduce financial risk). Thus IFFIm has provided around a third of Gavi’s funding to date.^1^ Gavi also receives funding (US$ 1.5 billion) from the advanced market commitment, which was an agreement by Gavi donors to pay for the creation of a new pneumococcal vaccine.^3^

**Table 1:** Pledged donations to the International Finance Facility for Immunisation, 2006–2014

Country	Cumulative pledges 2006–2014, in US$, millions (%)	Timescale, years	New pledges received in 2015, in US$, millions
Australia	256 (3.9)	20	0^a^
Brazil^b^	20 (0.3)	–	0
France	1899 (29.0)	20	180
Italy	635 (9.7)	20	0
Netherlands	186 (2.8)	12	72
Norway	264 (4.0)	15	0
South Africa	20 (0.3)	20	0
Spain	240 (3.7)	20	0
Sweden	38 (0.6)	15	0
United Kingdom	2980 (45.6)	23	0
**Total**	**6538 (100)**	–	**252**

IFFIm: International Finance Facility for Immunisation; US$: United States dollars.

^a^ Australia may use IFFIm for part of its new US$ 206 million donation to Gavi, the Vaccine Alliance.

^b^ Brazil’s commitment is awaiting final approval.

Note: Inconsistencies arise in some values due to rounding.

In the January 2015 pledging event to secure funds for Gavi for 2016–2020, Gavi requested US$ 1 billion to be committed through IFFIm. However, only US$ 252 million of new commitments were made by France and the Netherlands (Table 1).^4^ In contrast, Gavi received all of the US$ 7.5 billion it had requested through direct donations. The change in funding profile compared to the last round – i.e. reduction of funds pledged through IFFIm – has been described by credit ratings agencies as a result of “the diminishing policy importance of IFFIm for future financing of Gavi’s immunization programmes”.^5^

Here we evaluate stakeholders’ understanding and opinions of IFFIm. We also identify factors affecting funding levels and explore the future use of IFFIm at Gavi and for other issues in global health financing.

## Methods

This research has been conducted and reported in compliance with COREQ guidelines.^6^ We developed a topic guide using existing published literature on IFFIm and related technical documents for use within semi-structured interviews conducted in English (Box 1). The guide was piloted for suitability with three staff members within our department. Participants were not restricted to the questions and were allowed to discuss other topics freely.

Box 1 **Topic guide for the interview on stakeholders’ understanding and opinions of the International Finance Facility for Immunisation**

- Understanding of the role of IFFIm and the bond market mechanism.
- Perceived effectiveness and usefulness of IFFIm, particularly in relation to Gavi, the Vaccine Alliance.
- Views on factors affecting donors’ willingness to fund IFFIm.
- Views of any impact a reduction in IFFIm funding would have on Gavi.
- Views on future role for IFFIm both in relation to Gavi and more generally as a financing mechanism.

IFFm: International Finance Facility for Immunisation.

Ethical approval was received from the London School of Hygiene & Tropical Medicine Ethics Committee.

### Sampling

We initially identified 25 stakeholders using the criteria in Box 2 and invited them to voluntarily participate by email. Snowball sampling was used to identify other suitable interviewees, leading to a further 74 requests being made.

Box 2 **Criteria for identifying stakeholders of the International Finance Facility for Immunisation**

1. Stakeholders currently or historically involved with the function, administration or delivery of IFFIm were identified from document analysis of meeting attendance records.

2. Stakeholders from organizations who have donated to or received disbursements from IFFIm were identified from the IFFIm and Gavi, the Vaccine Alliance, websites.

3. Stakeholders from organizations representing people who have been beneficiaries of IFFIm funds were identified from the IFFIm and Gavi websites, especially the civil society organizations’ group.

4. Stakeholders who are currently doing research on financing global vaccine programmes or who have a historical research or policy interest in IFFIm were identified from publications in the academic and grey literature.

IFFm: International Finance Facility for Immunisation.

### Data collection and analysis

Between July and September 2015 we undertook 31 interviews and recorded 22.5 hours of material, each ranging from 15 minutes to 43 minutes, of which 28 were conducted via telephone and three face-to-face. Two interviews had two participants, and 29 had one participant. These were transcribed and uploaded into Nvivo v10 (QSR International, Cambridge, United States of America) for analysis using a framework method described elsewhere.^7^

One author coded all interviews and another author reviewed a sample of transcripts for accuracy. We categorized inductive and deductive codes by using a modified PESTLE framework.^8^ Results are reported using the four factors – that is, ideas, actor power, political contexts and issue characteristics – determining political priority described in the Shiffman and Smith 2007 framework.^9^

## Results

Of the total 99 invited, 41 declined – either due to not having the relevant expertise or because they referred us to a more suitable person in their organization – and 25 did not respond. Individuals from all major stakeholder organizations participated, except the World Bank, which declined. We grouped organizations into categories to preserve the confidentiality of individual participants.

The final sample consisted of 33 participants from 25 different organizations. Eight were associated with national government agencies and five were associated with nongovernmental organizations (NGOs). Both public–private partnership organizations and intergovernmental agency groups had four participants each. Three participants came from the pharmaceutical industry and two from academic institutions. Seven participants were not categorized to any group since they either no longer worked for an IFFIm stakeholder organization or worked for a specific industry that may make a participant identifiable if categorized separately.

### Ideas

Respondents expressed varying levels of understanding of IFFIm as a mechanism to fund vaccine programmes. Participants from NGOs and, to a lesser extent, government agencies, stated they lacked understanding. One NGO participant said:

“Personally I would really enjoy the ability to understand more about IFFIm and be able to speak about it intelligently and to explain … what it is and why it’s advantageous …”

Despite the mixed level of comprehension, almost all participants felt that IFFIm had been successful, particularly in its ability to raise money. The long-term nature of the funding was highly valued, as was the ability to front-load funds by making cash available up-front through accessing capital markets. This statement was supported by several comments that IFFIm provided Gavi with a stable, secure and flexible cash flow that has helped provide security between procurement and pledging cycles.

Other perceived successes mentioned were IFFIm’s role in increasing the visibility of demand and enabling Gavi to secure reduced prices. Participants from across the organizational categories saw IFFIm funds as an important contributing factor in Gavi being able to scale up coverage of vaccine programmes. However, this success was often discussed alongside the difficulty of separating out IFFIm’s contribution from Gavi’s work more generally, due to the pooling of funds.

### Actor power

#### Policy community cohesion

Participants held differing views about the future role of IFFIm. Many participants thought that IFFIm should continue to provide funds for Gavi, which were often expressed as a complementary mechanism to direct donations by providing a stable, predictable pool of cash to fund existing programmes. While other participants saw the on-going role for IFFIm as being ready to generate funds to finance new vaccines – such as Ebola and malaria vaccines. Several people stated that they felt IFFIm had served its purpose to capitalize Gavi in its start-up phase and should now slowly be phased out after meeting its bond commitments.

Several commented that a reduction in IFFIm funds would have negative impacts on Gavi, including: reduced funding predictability and potential for cash flow problems; increased risk of a slower response to emerging vaccine issues; reduced ability to deliver vaccine programmes; and an adverse effect on vaccine prices. However, others thought it would have no impact, largely because Gavi had been fully replenished through direct donations.

Three participants from donor governments said that IFFIm was seen as maintaining an older way of providing official development assistance. Lately, however, donor governments have emphasized the need for recipient countries to assume more responsibility for their spending on health.

#### Leadership and institutions

The IFFIm board was generally seen as effective at managing the bond financing mechanism. Historically, the British Department for International Development had led the creation and establishment of IFFIm and has been its biggest funder to date. However, participants saw the department’s policy as having changed from using IFFIm to fund Gavi, to now giving donations directly to Gavi, thus affecting IFFIm funding levels. Participants hypothesized this was due to the availability of funds resulting from the increased commitment of the British Government to spend 0.7% of Gross National Income on international development. More broadly, however, participants thought the department had not maintained the political will to advocate for IFFIm.

#### Civil society mobilization

Participants from both NGOs and governments stated that Gavi had not actively advocated for pledges to be made through IFFIm around the time of the pledging meeting in January 2015. Participants from government agencies explained that securing the required long-term agreements was a burdensome process and they would be unlikely to spontaneously undertake this without additional support from Gavi or encouragement from the NGO sector.

### Political contexts

#### Policy windows

Participants described a clear policy window in 2006 that brought together actors to establish IFFIm, with a drive to scale up vaccine programmes to meet the millennium development goals. However, this window closed following the 2008 financial crisis and the subsequent change in the macro-economic environment. All participants discussed the profound impact the crisis had on the policies of national governments. One participant from an intergovernmental agency stated:

“I do wonder the extent to which the notion of innovative financing … particularly government bond-funded investments … are much less appealing now in 2015 than they [were] in 2006 through 2008 … primarily because of the financial crisis that everyone lived through …”

Donors were seen as less likely to pledge to IFFIm after the financial crisis, because of the implementation of fiscal austerity in many donor countries. Participants from governments also expressed a preference for not being locked into multi-year commitments.

Government participants expressed divergent views on their intention to fund IFFIm in the future. Some donor governments intended to continue to fund IFFIm at their current level, while others did not plan to make any future commitments. Two governments were keen to increase their contributions and one was interested in pledging to IFFIm in the future, but had not done so in the past. Additionally, some participants thought that the likely global pool of donors had been saturated, particularly as budgetary cycles in Japan and the USA prevent long-term commitments.

#### Global governance structure

The nature of global vaccine finance has changed over time, particularly as Gavi has become a more established organization. Participants described Gavi as now being independently successful and leading the vaccine policy agenda. As a result, donors now prefer to donate directly rather than through IFFIm, which participants highlighted by the fact that Gavi was fully replenished. One participant from a public–private partnership said:

“They’ve been a victim of their own success … countries like giving money to Gavi and … historically Gavi’s ended up slightly overfunded … and when it has been overfunded … there’s no point borrowing out of IFFIm because … the money [is] in [its] own bank account.”

### Issue characteristics

Many participants discussed the important role IFFIm funds had played in enabling Gavi to scale up vaccine programmes in low-income countries. However, overall participants expressed doubt about whether IFFIm continued to be relevant.

Many felt that they did not have enough information on IFFIm’s performance. Some commented that they had not seen an evaluation. These comments were especially common among participants from NGOs and pharmaceutical industries, and to a lesser extent among the government participants. Those who were familiar with the independent 2011 evaluation^2^ felt that the report may need to be updated or its findings better communicated to stakeholders.

Several participants expressed uncertainty or concern about the costs of the management and administration of raising funds through the bond markets. While others thought that these costs were relatively low, all questioned whether this was cost–effective. Some participants, including from governments, discussed the complications arising from the downgrade of countries’ credit ratings and the subsequent impact on cost of borrowing from the capital markets.

### Bond financing

Many participants felt that bond financing could be beneficial for other global health or development issues. The most common proposals included: raising funds swiftly from pledges made in the face of emergency disease epidemics or in the event of a disaster; for the procurement of commodities such as drugs, technologies or bednets, which was framed both as an incentive for research and development, but also to provide security to companies producing the items; or to fund eradication programmes for specific diseases. However, it was pointed out that eradication programmes might not be an ideal candidate as they often have long, expensive end phases to eradicate the final cases (e.g. polio).

Other potential proposals mentioned were climate change and education, but many comments were sceptical, as the participants perceived the required interventions to be unclear or controversial. Water and sanitation were discussed more favourably, particularly to fund the initial infrastructure of pumps and pipes, while noting that these would require long-term funding for maintenance.

## Discussion

In the interviews stakeholders described changes related to each of the four factors that affect whether a global health issue, like financing vaccine programmes, is considered a political priority. First, IFFIm now exists within a different political context following the 2008 financial crisis and the effect this had on the financial position of donor governments. Second, in terms of the important ideas relating to IFFIm, stakeholders expressed uncertainty about the proposed benefits. Third, the characteristics of the issue have changed, with scaling up vaccine programmes using IFFIm funds seen as less of a priority now than in 2006, particularly as the cost–benefit trade-off of raising funds through IFFIm is not well understood by stakeholders. Finally, the power of actors has changed in relation to IFFIm, with disagreements identified among participants on the future use of IFFIm to raise funds and a reduced interest from civil society groups. Together, the changes described by stakeholders in relation to the four factors provide a possible explanation why there were fewer commitments in the January 2015 pledging conference.

The participants had divergent views about IFFIm’s on-going role. Overall IFFIm was seen as having been successful in a wide variety of ways, including accessing new funds and influencing the vaccine market, which have led to an expectation that IFFIm will continue in a similar role. IFFIm also provides security and confidence to the pharmaceutical industry, as the cost of delivering vaccine programmes is likely to increase, since the cost has already risen with the addition of new vaccines from US$ 0.67 in 2001 to US$ 45.59 in 2014.^10^ Using IFFIm funds to smooth out the procurement cycle reduces Gavi’s dependence on the receipt of donated funds, which is known to cause difficulties in other similar organizations, such as ensuring timely payment for supply of goods.^11^ However, participants highlighted that Gavi was fully replenished through direct donations in its most recent funding round, suggesting additional front-loaded funds may not be required. Since Gavi did not request any funds from IFFIm in 2014 there is currently a surplus in IFFIm,^1^ leading some participants to question IFFIm’s future relevance.

Participants were also unsure of the financing and management costs. At inception, most donor countries had the highest credit ratings (AAA), however some ratings have been successively downgraded, which could make raising funds through bond issuances more difficult or expensive.^12^ IFFIm has not experienced such problems since it continues to issue bonds at competitive rates, although this has not been communicated well to stakeholders. However, if AAA rated donors stop funding IFFIm, the costs of issuing bonds with only low-rated donors will be much higher. If funding is to be maintained, then IFFIm and Gavi will need to provide additional evidence of IFFIm’s cost–benefit trade-off and be sensitive to the differing circumstances and priorities of government donors.

IFFIm-like mechanisms have been proposed to fund a wide range of other global health issues, including malaria control, Ebola vaccine and noncommunicable diseases.^13^^–^^15^ More broadly, IFFIm has also been proposed as a possible mechanism to raise funds to meet the sustainable development goals and the outcome document for the Third International Conference on Financing For Development, explicitly encourages the development of IFFIm-like mechanisms.^16^^,^^17^ There are several unique features about vaccine programmes that could make transferability of an IFFIm-like mechanism to other areas challenging.^12^ However, participants highlighted two circumstances where the use of an IFFIm-type mechanism may be appropriate.

First, bond financing could have a role in emergency preparedness, including disasters and pandemics. In the context of a pledging conference to urgently raise funds for a natural disaster or infectious disease outbreak, an IFFIm-like mechanism could be used to generate the cash from donor pledges relatively quickly rather than waiting for them to be mobilized over time. A recent study found that of the US$ 2.89 billion pledged to combat the 2013–2016 Ebola virus disease outbreak, only US$ 1.09 billion had been collected by mid-2015.^18^ If bond financing had been used, funds might have been available closer to the time when they were most needed.

Second, bond financing could support quantifiable, outcomes-based, time-limited interventions, including the formation of a new organization or delivery of a specific intervention, for example a catch-up vaccination programme. The risk of using front-loaded funds for programmes with on-going costs – such as maintenance or staffing – is that the benefit of an increase in infrastructure is negated by its deterioration or disrepair without sustainable funding once the initial funds have been spent.

Any new bond financing initiative should have well-defined objectives and quantifiable outcomes to ensure that its cost–effectiveness can be evaluated. One example of this is when organizations release social impact bonds which are bonds sold to investors to generate funds for development projects that have clear evaluation criteria and are highly outcomes focused.^19^ Another example is the development impact bonds released by the British Department for International Development to fund African sleeping sickness prevention programmes.^20^ Unlike IFFIm bonds, investors are only repaid if the programme funded is successful. This increases the risk to bondholders of not being repaid, but also increases buy-in from the private sector organizations that buy the bonds, who are motivated to ensure the programmes are successful.^21^

This study has limitations. The sample may suffer from volunteer bias and is unlikely to cover the full range of views relating to IFFIm. The World Bank declined, as did a small number of government agencies, notably those from low-income countries. Several NGOs, both international and local, could not identify a relevant member of staff to participate.

In conclusion, IFFIm is unique in international development finance and is seen as successful by many stakeholders. However, the benefit of pledging funds through IFFIm needs to be re-evaluated and communicated to stakeholders. The IFFIm financing mechanism has the potential to raise funds for other global health issues. However these issues must be carefully considered as to whether bond financing could be effective and must have quantifiable pre-defined outcome indicators to evaluate performance.
