# Corrigendum

**DOI:** 10.2471/BLT.16.121016

**Published:** 2016-10-01

In Volume 94, Issue 9, September 2016, page 687, the fourth, fifth and sixth sentences of the third paragraph should read: "The description of the average annual administration costs of IFFIm as $115m per year is inaccurate. On average between 2010 and 2014 the true operating costs of IFFIm were $5m per year. The remaining $110m was the average annual financing costs of the bonds, including interest payments, which depend on the terms of each transaction such as issue size and maturity, and not incurred as part of IFFIm’s operating costs."

